# An experimental study of exenatide effects on renal injury in diabetic rats^1^

**DOI:** 10.1590/s0102-865020190010000001

**Published:** 2019-02-14

**Authors:** Xiaodong Wang, Zhaoliang Li, Xiaolei Huang, Fenghua Li, Jinbo Liu, Zhenzuo Li, Dongfang Bai

**Affiliations:** I Master, Second Department of Nephrology, Tai’an Central Hospital, China. Technical procedures, critical revision, final approval.; II Bachelor, Second Department of Endocrinology, Tai’an Central Hospital, China. Technical procedures, statistical analysis, critical revision, final approval.; III Master, Department of Hemodialysis, Tai’an Central Hospital, China. Acquisition of data, critical revision, final approval.; IV MD, Department of Endocrinology, Qilu Hospital, Shandong University, China. Statistical analysis, critical revision, final approval.; V MD, Department of Endocrinology, The Fourth People’s Hospital of Ji’nan City, China. Manuscript writing, critical revision, final approval.; VI Master, Second Department of Endocrinology, Tai’an Central Hospital, China. Design of the study, critical revision, final approval.

**Keywords:** Exenatide, Diabetes Mellitus, Acute Kidney Injury, Oxidative Stress, Rats

## Abstract

**Purpose:**

To investigate the effects of exenatide on renal injury in streptozotocin-induced diabetic rats.

**Methods:**

Fifty SD rats were randomly divided into normal control, model, exenatide-1, exenatide-2 and exenatide-3 groups, 10 rats in each group. The diabetic nephropathy model was constructed in later 4 groups. Then, the later 3 groups were treated with 2, 4 and 8 μg/kg exenatide for 8 weeks, respectively. The serum and urine biochemical indexes and oxidative stress and inflammatory indexes in renal tissue were determined.

**Results:**

Compared to the model group, in exenatide-3 group the serum fasting plasma glucose and hemoglobin A1c levels were significantly decreased, the fasting insulin level was significantly increased, the renal index and blood urea nitrogen, serum creatinine and 24 h urine protein levels were significantly decreased, the renal tissue superoxide dismutase and glutathione peroxidase levels were significantly increased, the malondialdehyde level was significantly decreased, and the renal tissue tumor necrosis factor alpha, interleukin 6, hypersensitive C-reactive protein and chemokine (C-C motif) ligand 5 levels were significantly decreased P<0.05).

**Conclusions:**

Exenatide can mitigate the renal injury in diabetic rats. The mechanisms may be related to its resistance of oxidative stress and inflammatory response in renal tissue.

## Introduction

 Chronic kidney disease is one of the most important diseases threatening the human health. With the improvement of living standards and the aging of the population, the diabetic nephropathy has gradually become the primary cause of chronic kidney disease, surpassing the chronic glomerulonephritis. Diabetic nephropathy is one of the most common microvascular complications of diabetes mellitus, and is one of the main causes of chronic renal failure and death of diabetes mellitus^1^. At present, the main direction for treatment of diabetic nephropathy is controlling blood glucose and blood pressure and reducing urinary protein, but the effect is not satisfactory^2^. The pathogenesis of diabetic nephropathy is complex. It is believed that the diabetic nephropathy is related to many factors, such as renal vascular dynamic changes, oxidative stress, inflammatory reaction, glucose metabolism disorder, etc.^3^ ^-^ ^5^. These factors often interact with each other and promote the process of diabetic nephropathy together. Exenatide, a synthetic incretin-mimetic peptide, is currently considered an attractive agent for the treatment of diabetes mellitus. It has biological properties similar to human glucagon-like peptide-1 (GLP-1), a regulator of insulin secretion and glucose metabolism. Exenatide shares approximately 53% homology with the mammalian incretin GLP-1 and binds to and activates GLP-1 receptor cloned from islet cells, gut, hypothalamus, and kidney^6^. It is found that, in addition to lowering blood glucose, exenatide has anti-oxidative stress, anti-inflammatory and apoptosis inhibitory effects^7^ ^-^ ^9^. This study investigated the effects of exenatide on renal injury in diabetic rats and explored the related mechanisms. The objective was to provide an experimental basis for clinical application of exenatide to prevention and treatment of diabetic nephropathy.

## Methods

 This study was performed in strict accordance with the recommendations in the Guide for the Care and Use of Laboratory Animals of the National Institutes of Health. The animal use protocol has been reviewed and approved by the Institutional Animal Care and Use Committee of Tai’an Central Hospital. 

###  Animal grouping and modeling 

 Fifty male Sprague Dawley rats (200±30 g) were adaptively fed for 1 week. Then, the rats were divided into normal control, model, low-dose exenatide (exenatide-1), middle-dose exenatide (exenatide-2) and high-dose exenatide (exenatide-3) groups according to random number table, with 10 rats in each group. The rats in model and 3 exenatide groups were fasted for 12h, followed by single sterile intraperitoneal injection of streptozotocin with dose of 60 mg/kg. After 72h, the tail vein blood was sampled, and the fasting blood glucose (FBG) level higher than 16.7 mmol/L indicated the diabetes. After 3 weeks, the 24-hours urine protein (24h UP) was detected. The 24h UP higher than 30 mg indicated the diabetic nephropathy^10^. In this study, the diabetic nephropathy model was successfully constructed in 40 rats.

###  Treatment methods 

 After establishment of diabetic nephropathy model, the rats in exenatide-1, exenatide-2 and exenatide-3 groups were subcutaneously injected with exenatide (Baxter Pharmaceutical Solutions LLC, YN, USA), with dose of and 2, 4 and 8 μg/kg, respectively. The normal control and model groups were subcutaneously injected with equal volume of solvent. The injection was performed once per day, and was lasted for 8 weeks. During the treatment, the general conditions of rats were observed. After treatment, the 24-hours urine samples were collected. The body weight of rats was measured. After fasting for 12 h, the rats were anesthetized with chloral hydrate. A 10 ml blood was collected from the abdominal aorta, and was kept for test. The left and right kidneys were taken out quickly. After removing the capsules, the kidneys were weighed. The renal index (kidney weight/body weight, mg/g) was calculated. The kidneys were stored at -80^o^C for later determination.

###  Determination of serum and urine biochemical indexes 

 The blood samples were centrifuged at 2000 r/min for 10 min to obtain the serum. The FGB and fasting insulin (FINS) levels were detected according to the kit instructions. The hemoglobin A1c (HbA1c) was detected by enzyme linked immunosorbent assay. The blood urea nitrogen (BUN), serum creatinine (Scr) and 24h UP levels were measured by automatic biochemical analyzer. The kits were provided by Sigma-Aldrich Corp. (MO, USA).

###  Determination of oxidative stress and inflammatory indexes in renal tissue 

 The kidneys of rats were taken, and the 10% renal tissue homogenate was made from 100 mg renal tissue using 5 ml Tris-HCl solution (pH 7.4). After centrifugation at 2000 r/min for 10 min, the supernatant was obtained. The superoxide dismutase (SOD) level was determined using WST-1 method^11^. The glutathione peroxidase (GSH-Px) level was determined by colorimetric method^12^. The malondialdehyde (MDA) level was determined by TBA method^13^. The procedures were in accordance to the instructions of kits. The tumor necrosis factor alpha (TNF-α), interleukin 6 (IL-6), hypersensitive C-reactive protein (hs-CRP) and chemokine (C-C motif) ligand 5 (CCL5) levels were determined using enzyme-linked immunosorbent assays. The kits were provided by Sigma-Aldrich Corp. (MO, USA).

###  Statistical analysis 

 The data were analyzed using SPSS 22.0 software (SPSS Inc., IL, USA). The data were presented as mean±standard deviation. The differences among different groups were analyzed using one-way analysis of variance, followed by pairwise comparison using SNK-q test. A P < 0.05 was accepted as statistically significant.

## Results

###  General condition of rats 

 During the treatment period, the rats in normal control group were in good condition, with glossy fur, free movement and sensitive reaction. In model group, the rats had obviously poor mental state. The fur gradually lost luster. The movement and response were slow. Compared with model group, the symptoms of rats in 3 exenatide groups were mild, especially in exenatide-2 and exenatide-3 groups. There was no rat dying in each group during the experiments.

###  Effects of exenatide on body weight of rats 

**Figure 1:**
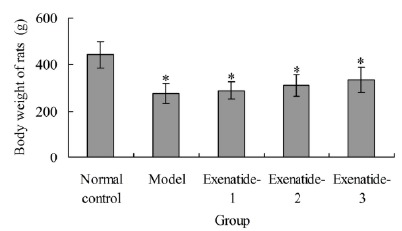
Body weight of rats in different groups.

 At the end of treatment, the body weight of rats in normal control group was 442.34±58.12 g. The body weight in model, exenatide-1, exenatide-2 and exenatide-3 groups were 277.62±42.33 g, 288.14±36.29 g, 311.58±45.48 g and 334.04±57.46 g, respectively, which was significantly lower than normal control group, respectively (P<0.05). There was no significant difference of body weight among model group and 3 exenatide groups (P>0.05) (Figure 1). 

*P<0.05 compared with normal control group.

###  Effects of exenatide on glucose metabolism indexes of rats 

 After treatment, compared with the normal control group, in model group and 3 exenatide groups the FBG and HbA1 levels were significantly increased, respectively (P < 0.05), and the FINS level was significantly decreased, respectively (P < 0.05). Compared with the model group, the FBG and HbA1 levels in exenatide-2 and exenatide-3 groups were significantly decreased, respectively (P < 0.05), and the FINS level in exenatide-3 group was significantly increased (P < 0.05) (Table 1).

**Table 1:** Glucose metabolism indexes of rats in different groups.

Group	FBG (mmol/L)	FINS (μg/L)	HbA1 (%)
Normal control	5.52±1.06	0.88±0.12	4.33±0.74
Model	20.33±3.12^*^	0.55±0.08^*^	10.48±1.85^*^
Exenatide-1	19.48±2.87^*^	0.56±0.07^*^	10.08±1.72^*^
Exenatide-2	17.36±2.73^*#%^	0.62±0.08^*^	8.12±1.98^*#%^
Exenatide-3	17.04±2.56^*#%^	0.73±0.09^*#%&^	8.05±1.44^*#%^

^*^P < 0.05 compared with normal control group; ^#^P < 0.05 compared with model group; ^%^P < 0.05 compared with exenatide-1 group; ^&^P < 0.05 compared with exenatide-2 group. FBG, fasting plasma glucose; FINS, fasting insulin; HbA1c, hemoglobin A1c.

###  Effects of exenatide on renal function indexes of rats 

Table 2 showed that, after treatment, compared with the normal control group, the renal index and Scr level in mode, exenatide-1 and exenatide-2 groups and BUN and 24 h UP levels in model and 3 exenatide groups were significantly increased, respectively (P < 0.05). Compared with the model group, in exenatide-2 and exenatide-3 groups the renal index, BUN, Scr and 24 h UP levels were significantly decreased, respectively (P < 0.05).

**Table 2:** Renal function indexes of rats in different groups.

Group	Renal index (mg/g)	BUN (mmol/L)	Scr (μmol/L)	24h UP (mg)
Normal control	1.41±0.17	6.82±1.45	22.46±2.58	6.47±1.26
Model	2.18±0.22^*^	11.52±1.98^*^	28.83±3.07^*^	62.73±11.73^*^
Exenatide-1	2.11±0.28^*^	10.77±1.46^*^	27.28±2.72^*^	58.29±10.63^*^
Exenatide-2	1.92±0.26^*#%^	9.02±1.07^*#%^	25.78±2.62^*#%^	42.63±8.51^*#%^
Exenatide-3	1.61±0.23^#%&^	8.82±1.97^*#%^	24.19±2.62^#%&^	34.68±7.27^*#%&^

^*^P < 0.05 compared with normal control group; ^#^P < 0.05 compared with model group; ^%^P < 0.05 compared with exenatide-1 group; ^&^P < 0.05 compared with exenatide-2 group. BUN, blood urea nitrogen; Scr, serum creatinine; 24h UP, 24-hour urine protein.

###  Effects of exenatide on renal tissue oxidative stress indexes of rats 

 After treatment, compared with the normal control group, in mode, exenatide-1 and exenatide-2 groups the renal tissue SOD and GSH-Px levels were significantly decreased, respectively (P < 0.05), and the renal tissue MDA level was significantly increased, respectively (P < 0.05). Compared with model group, the SOD level in exenatide-2 and exenatide-3 groups was significantly increased, respectively (P < 0.05), the GSH-Px level in exenatide-3 group was significantly increased (P < 0.05), and the MDA level in exenatide-2 and exenatide-3 groups was significantly decreased, respectively (P < 0.05) (Table 3). 

**Table 3:** Renal tissue oxidative stress indexes in different groups.

Group	SOD (U/mg prot)	GSH-Px (U/mg prot)	MDA (nmol/mg prot)
Normal control	193.36±21.32	25.44±3.28	0.79±0.57
Model	133.59±15.34^*^	18.12±2.56^*^	1.25±0.21^*^
Exenatide-1	136.17±16.12^*^	18.67±3.12^*^	1.23±0.15^*^
Exenatide-2	156.66±19.89^*#%^	19.47±4.04^*^	1.05±0.19^*#%^
Exenatide-3	178.131±19.71^#%&^	23.72±3.56^#%^	0.98±0.22^#%^

^*^P < 0.05 compared with normal control group; ^#^P < 0.05 compared with model group; ^%^P < 0.05 compared with exenatide-1 group; ^&^P < 0.05 compared with exenatide-2 group. SOD, superoxide dismutase; GSH-Px, glutathione peroxidase; MDA, malondialdehyde.

###  Effects of exenatide on renal tissue inflammatory indexes of rats 

 As shown in Table 4, after treatment, compared with the normal control group, in model group and 3 exenatide groups the renal tissue TNF-α, IL-6, hs-CRP and CCL5 levels were significantly increased, respectively (P < 0.05). Compared with the model group, the renal tissue IL-6 level in exenatide-1, exenatide-2 and exenatide-3 groups was significantly decreased, respectively (P < 0.05), and the renal tissue TNF-α, hs-CRP and CCL5 levels in exenatide-2 and exenatide-3 groups were significantly decreased, respectively (P < 0.05).

**Table 4:** Renal tissue inflammatory indexes in different groups.

Group	TNF-α (ng/L)	IL-6 (ng/L)	hs-CRP (mg/L)	CCL5 (ng/L)
Normal control	67.44±11.06	78.29±10.73	38.46±6.12	55.46±7.78
Model	113.37±14.67^*^	125.36±10.37^*^	67.84±8.44^*^	98.84±9.84^*^
Exenatide-1	109.21±15.32^*^	108.63±11.29^*#^	63.63±7.85^*^	90.63±9.12^*^
Exenatide-2	95.56±10.14^*#^	102.12±14.83^*#^	56.72±7.13^*#^	76.72±8.06^*#%^
Exenatide-3	79.73±9.86^*#%&^	93.29±9.18^*#%^	47.39±6.36^*#%^	69.39±7.39^*#%^

^*^P < 0.05 compared with normal control group; ^#^P < 0.05 compared with model group; ^%^P < 0.05 compared with exenatide-1 group; ^&^P < 0.05 compared with exenatide-2 group. TNF-α, tumor necrosis factor alpha; IL-6, interleukin 6; hs-CRP, hypersensitive C-reactive protein; CCL5, chemokine (C-C motif) ligand 5.

## Discussion

 GLP-1 is one of the important intestinal regulatory hormones in the homeostasis of blood glucose. On the one hand, GLP-1 can enhance the glucose-dependent insulin secretion response. On the other hand, it can promote the satiety and reduce food intake by acting on the central nervous system, thereby reducing the burden of islet beta cells and lowering the body weight. At present, GLP-1 is one of the hotspots in the research of hypoglycemic drugs^14^. Exenatide, the GLP-1 analogue, has the effects similar with endogenous GLP-1. It can bind to GLP-1 and activate its receptor^6^. In the present study, the rat diabetic nephropathy model was constructed, and the effects of exenatide on renal injury in rats were investigated. Results showed that, compared with the model group, the FBG and HbA1 levels in exenatide-2 and exenatide-3 groups were significantly decreased, and the FINS level in exenatide-3 group was significantly increased. This is basically consistent with previous reports^15^ ^,^ ^16^. In addition, compared with the model group, in exenatide-2 and exenatide-3 groups the renal index, BUN and Scr levels and 24 h UP level were significantly decreased. This indicates that, besides lowering blood glucose, exenatide can reduce the renal injury of diabetic rats.

 Oxidative stress plays an important role in the development of diabetic nephropathy^17^. SOD is an important enzyme widely existing in the body. The content of SOD reflects the ability of scavenging free radicals^18^. GSH-Px is a peroxidase which can protect the structure and function of cell membrane from peroxide interference and damage^19^. When the renal injury occurs, a large number of oxygen free radicals will generate and accumulate, which leads to the lipid peroxidation. MDA is a product of lipid peroxidation, and its content represents the degree of lipid peroxidation^20^. Results of this study showed that, compared with the normal control group, in model group the renal tissue SOD and GSH-Px levels were significantly decreased, and the renal tissue MDA level was significantly increased. Compared with model group, the SOD level in exenatide-2 and exenatide-3 groups was significantly increased, the GSH-Px level in exenatide-3 group was significantly increased, and the MDA level in exenatide-2 and exenatide-3 groups was significantly decreased. This indicates that, the oxidative stress is related to the renal injury of diabetic rats, and exenatide has the ability of scavenging radical and reducing lipid peroxidation, thus playing a role in alleviating the renal injury.

 TNF-α is a pro-inflammatory factor with negative inotropic action, and is the initiation factor of the inflammatory cascade reaction. It can induce glomerular vascular endothelial cells to secrete adhesion factors, promote the proliferation of glomerular mesangial cells, and induce the glomerular lesions^21^. IL-6 is a pro-inflammatory cytokine. After stimulation by some antigens, the mesangial cells can sustainably secrete IL-6. Thus the serum IL-6 level is significantly increased. The increased IL-6 can stimulate the proliferation of mesangial cells, induce the pathological changes of glomeruli and abnormal structure and function^22^. In addition, IL-6 can stimulate the hepatocytes to produce a large number of hs-CRP, which induces or aggravates the inflammatory response^23^. CCL5, a secretory protein, is a member of the chemokine CC family. It participates in the immune regulation and inflammation process. The kidney, fibroblasts, adipocytes, corneal stromal cells, platelets and other cells can produce CCL5. CCL5 can activate and induce the recruitment of monocytes and macrophages, thereby inducing the release of inflammatory factors and aggravating the inflammatory response^24^. In the present study, compared with the normal control group, the renal tissue TNF-α, IL-6, hs-CRP and CCL5 levels in model group were significantly increased. Compared with the model group, the levels of these indexes exenatide-3 group were significantly decreased. This suggests that, the inflammatory response is involved in the renal injury in diabetic rats. Exenatide can reduce the inflammatory response, thus reducing the renal injury.

## Conclusions

 The exenatide can mitigate the renal injury in diabetic rats. The mechanisms may be related to its resistance of oxidative stress and inflammatory response in renal tissue. This study has provided an experimental basis for clinical application of exenatide to prevention and treatment of diabetic nephropathy. There are still some limitations in this study. Firstly, the correlations among different indexes are not investigated. Secondly, maybe there are other mechanisms of exenatide in alleviating renal injury.
